# Risk Assessment of Diagnostic Categories in the Proposed Sydney System for Reporting Lymph Node Cytopathology: A Retrospective Cytomorphological Study

**DOI:** 10.30699/ijp.2025.2058284.3445

**Published:** 2025-10-15

**Authors:** Surabhi, Avinash Singh, Shambhawi Sharma, Tarun Kumar, Bhadani Punam Prasad, Shreekant Bharti, Ruchi Sinha

**Affiliations:** 1 Department of Pathology, All India Institute of Medical Sciences, Patna, Bihar, India

**Keywords:** fine-needle aspiration, lymphadenopathy, malignancy, retrospective, diagnostic accuracy

## Abstract

**Background & Objective:**

Fine-needle aspiration cytology (FNAC) is a minimally invasive, rapid, and relatively safe diagnostic method for the initial evaluation of lymphadenopathy of unknown origin. In May 2020, the Sydney System was proposed to provide recommendations for diagnostic categorization, FNAC of lymphadenopathy, pathology reporting, and related practices. This study aimed to analyze the applicability of the Sydney System in lymph node FNAC and to evaluate diagnostic accuracy and risk of malignancy (ROM) for each diagnostic category.

**Methods:**

A 2-year retrospective diagnostic study was conducted from January 2019 through December 2020. Sensitivity, specificity, positive predictive value, negative predictive value, diagnostic accuracy (DA), and ROM were calculated using histopathology as the gold standard.

**Results:**

A total of 632 lymph node FNAC cases were included, with histopathological follow-up available in 45 cases. The median age of patients was 26 years, with a male-to-female ratio of 1.2:1. Cervical lymph nodes were most frequently involved (367 cases, 58.1%). Overall sensitivity, specificity, and diagnostic accuracy were 66.7%, 85.0%, and 76.3%, respectively. ROM by diagnostic category was: nondiagnostic (25%), benign (5.2%), atypia of undetermined significance (AUS) (50%), suspicious (80%), and malignant (88.8%).

**Conclusion:**

FNAC demonstrated high diagnostic efficacy when applied using the proposed Sydney System, supporting the utility of this standardized reporting scheme for lymph node cytology.

## Introduction

Fine-needle aspiration cytology (FNAC) is an essential diagnostic tool for the initial evaluation of lymphadenopathy of unknown origin. It is minimally invasive, efficient, rapid, and reliable, and it also provides material for ancillary techniques that enhance diagnostic accuracy (1). In India, the incidence of lymph node malignancies ranges from 65.7% to 80.4% and is often metastatic in nature, while lymphomas account for 2% to 15.3% of aspirated lymph nodes across all sites (2,3).

The diagnosis of lymph node enlargement may be straightforward in cases of infections or metastatic carcinomas but is often more complex in lymphomas (4). A small percentage of lymph node disorders remain undiagnosed by FNAC, necessitating biopsy. Beyond histopathology, the diagnosis of lymphomas increasingly relies on a combination of cytomorphological features, flow cytometry, cytogenetics, and molecular studies, as reflected in the most recent WHO classification of lymphomas (5).

Adherence to proper technique is critical in lymph node FNAC. Rapid on-site evaluation (ROSE) has been shown to improve diagnostic yield, reduce costs, and facilitate triaging for the cost-effective selection of ancillary tests (6,7).

In cytopathology, several standardized reporting systems such as Bethesda, Milan, and Yokohama are widely used and accepted by cytopathologists and clinicians. To ensure consistency in lymph node cytology reporting, a dedicated classification system was required. In May 2020, the Sydney System was proposed to address this need, offering recommendations for diagnostic categorization, lymph node FNAC procedures, pathology reporting, and related guidelines (8). The system defines five diagnostic categories based on distinct cytomorphological features: category I/L1, inadequate or nondiagnostic; category II/L2, benign; category III/L3, atypical cells of undetermined significance or atypical lymphoid cells of uncertain significance; category IV/L4, suspicious; and category V/L5, malignant (9).

Despite its potential, the Sydney System has not yet been fully implemented, and the available literature remains limited. The present study was therefore undertaken to analyze the applicability of the Sydney System in lymph node FNAC and to evaluate its diagnostic accuracy and the associated risk of malignancy (ROM) for each diagnostic category.

## Materials and Methods

A two-year retrospective diagnostic analytical study was conducted at a tertiary care center from January 2019 through December 2020 after obtaining clearance from the institutional ethics committee. The study included all patients presenting to the cytology clinic with enlarged lymph nodes. For cases in which FNAC was performed more than once, only the first diagnosis was considered. Noncooperative cases in which FNAC could not be performed were excluded.

Patient demographic data, local examination findings, and radiologic details were retrieved from the Medical Records Department (MRD) and hospital information system (HIS). FNAC was performed following the institute’s standard protocol. Rapid on-site evaluation (ROSE) was not used. An experienced cytopathologist performed FNAC using a 23-gauge needle, with a maximum of two passes per case. For larger swellings, aspirations were taken from multiple sites to minimize sampling error. Ultrasound guidance was used in selected cases.

Smears were prepared by spreading the aspirated material on glass slides. Half of the material was fixed in 90% ethanol for Papanicolaou staining, and the other half was air-dried for Giemsa staining. All slides were reviewed independently by two experienced cytopathologists. In cases of diagnostic discrepancy, the opinion of the senior cytopathologist was considered final. Each case was classified according to the proposed Sydney System into five categories. Tru-cut and excisional biopsy specimens underwent standard histopathological processing, and cytohistological correlation was performed. Diagnoses were based solely on cytomorphological evaluation of Papanicolaou- and Giemsa-stained smears; ancillary techniques such as flow cytometry and immunocytochemistry were not performed.

Quantitative data were expressed as means and standard deviations, while categorical variables were reported as frequencies and percentages. Sensitivity, specificity, positive predictive value, negative predictive value, and diagnostic accuracy (DA) were calculated using histopathology as the gold standard. Sensitivity was defined as the proportion of true-positive cases, and specificity as the proportion of true-negative cases. Diagnostic accuracy was calculated as the proportion of true-positive and true-negative cases among all evaluated cases. The risk of malignancy (ROM) for each diagnostic category was calculated as the number of malignant cases divided by the total number of cases in that category with histopathological follow-up.

## Results

A total of 632 cases of lymph node enlargement undergoing FNAC between January 2019 and December 2020 were included in the study. The median patient age was 26 years (range, 2–89 years). The male-to-female ratio was 1.2:1. The cervical group was the most frequently involved lymph node (58.1%), followed by inguinal (10.7%), axillary (9.1%), submandibular (8.0%), and supraclavicular (6.4%) groups. The greatest lymph node dimension ranged from 0.5 to 4.5 cm, with a mean of 1.8 cm (Table 1).

The distribution of cases according to the Sydney System is shown in Table 2. The majority fell into category L2 (benign), with 533 cases (84.3%). Among these, reactive lymphadenitis was the most frequent diagnosis (260 cases, 41.1%). Granulomatous lymphadenitis was the second most common diagnosis, with acid-fast bacilli positivity in 34 cases (5.3%). Category L1 (inadequate) included 12 cases (1.9%). Category L3 (atypia of undetermined significance [AUS]) comprised 21 cases (3.3%), all reported as lymphoproliferative disorders. Categories L4 (suspicious) and L5 (malignant) accounted for 9 cases (1.4%) and 57 cases (9.0%), respectively (Figures 1 and 2).

Histopathological follow-up was available for 45 cases (Table 3). Concordance was observed in 32 cases, while 13 were discordant. In category L1 (inadequate), 4 cases had follow-up; 1 was discordant, diagnosed as metastatic malignant melanoma on histopathology, while the others were tuberculous lymphadenitis (2) and reactive lymphoid hyperplasia (1). Among 19 L2 (benign) cases, only 1 was discordant, where peripheral T-cell lymphoma was misdiagnosed as Kimura disease on FNAC. Of 8 L3 (AUS) cases, only 1 showed concordance, reported as a lymphoproliferative disorder, with immunohistochemistry (IHC) advised but not performed. The remaining 7 cases were discordant: reactive lymphoid hyperplasia (3), anaplastic large cell lymphoma (2), Hodgkin lymphoma (1), and non-Hodgkin lymphoma (1). In category L4 (suspicious), 5 cases had follow-up. One was discordant, reported as suspicious for metastatic papillary thyroid carcinoma on cytology but found to be reactive lymphoid hyperplasia on histopathology. The remaining 4 were confirmed malignant: peripheral T-cell lymphoma (1), non-Hodgkin lymphoma (2), and Hodgkin lymphoma (1). Histopathology was available for 9 L5 (malignant) cases. One was discordant, diagnosed as non-Hodgkin lymphoma on FNAC but reactive lymphoid hyperplasia on histopathology. The remaining 8 were concordant: metastatic squamous cell carcinoma (3), metastatic adenocarcinoma (1), metastatic ductal carcinoma (1), and non-Hodgkin lymphoma (3) (Figure 3).

The overall sensitivity, specificity, positive predictive value, and negative predictive value of FNAC using the Sydney System were 66.7%, 85.0%, 80.0%, and 73.9%, respectively (Table 4). The risk of malignancy (ROM) for each diagnostic category is presented in Table 3, and diagnostic accuracy (DA) for each category is also summarized in Table 3.

**Table 1:** Demographics of 632 lymph node FNAC

Demographics		Number	Percentage
Sex	Female	288	45.5%
	Male	344	54.5%
Age	Median	26 years	
	Minimum	2 years	
	Maximum	89 years	
Location	Cervical group	367	58.1%
	Axillary	58	9.1%
	Submandibular	51	8%
	Submental	8	1.2%
	Inguinal	68	10.7%
	Supraclavicular	41	6.4%
	Abdominal	13	2%
	Pre and post auricular	21	3.3%
	Epitrochlear	4	0.6%
	Intraparotid	1	0.15%

Abbreviations. NHL, Non-Hodgkin’s Lymphoma; HL, Hodgkin’s Lymphoma

**Table 2:** Distribution of cases in various cytodiagnostic categories of Sydney system

Diagnostic Category	Cytopathological Diagnosis	Number of cases	Percentage
L1 Inadequate/non-diagnostic	Inadequate	12	1.9%
L2 Benign	Total cases	533	84.3%
	Reactive lymphadenitis	260	41.1%
	Granulomatous lymphadenitis (AFB positive)	228 (34)	36% (5.3%)
	Necrotizing lymphadenitis	31	4.9%
	Acute suppurative lymphadenitis	13	2%
	Kimura disease	1	0.1%
L3 AUS	Lymphoproliferative disorder	21	3.3%
L4 Suspicious	Total cases	9	1.42%
	HL	2	0.3%
	NHL	5	0.8%
	Metastasis	2	0.3%
L5 Malignant	Total cases	57	9%
	HL	5	0.8%
	NHL	13	2%
	Metastasis	39	6.1%

Abbreviations. NHL, Non-Hodgkin’s Lymphoma; HL, Hodgkin’s Lymphoma

**Figure:**
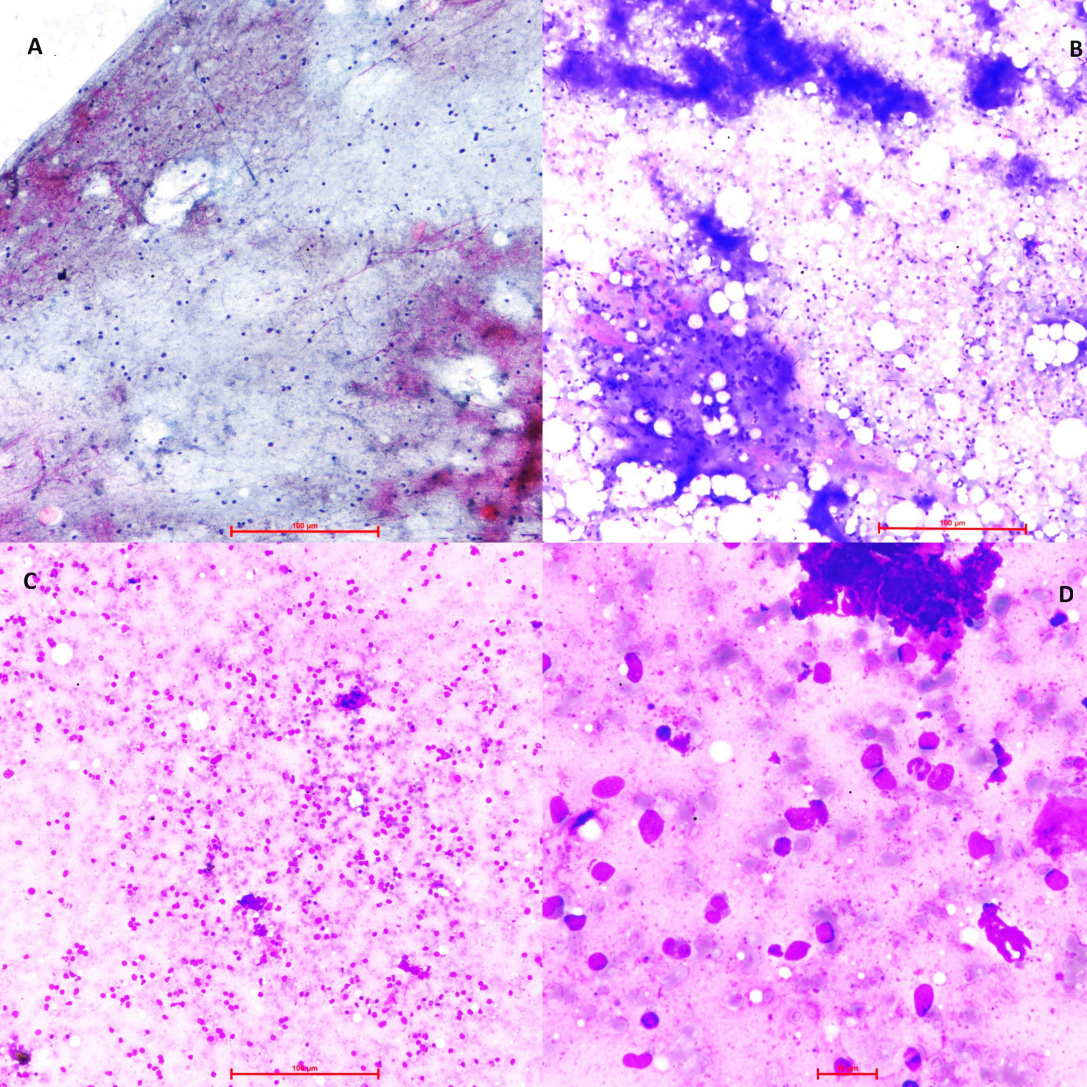
A: Microphotograph shows hemorrhage with occasional scattered lymphoid cells only. Non diagnostic category. (Pap,10x) B: Microphotograph shows well-formed granuloma. Benign Category. (Giemsa,10x) C: Microphotograph shows scattered atypical lymphoid cells. AUS category. ( Giemsa,20x) D: Microphotograph shows scattered atypical lymphoid cells. Background shows eosinophils. AUS category. (Giemsa, 40x)

**Table 3:** Cytohistological correlation in 45 cases with associated risk of malignancy and diagnostic accuracy in each category.

Diagnostic Categories	FNAC	Histopathology		Risk of malignancy (ROM) in each category	Diagnostic accuracy
		Histopathological follow up	Confirmed malignant lesion		
L1 Inadequate/non-diagnostic	12	4	1	25%	50%
L2 Benign	533	19	1	5.2%	93.75%
L3 AUS	21	8	4	50%	28.57%
L4 Suspicious	9	5	4	80%	80%
L5 Malignant	57	9	8	88.8%	87.5%

**Figure:**
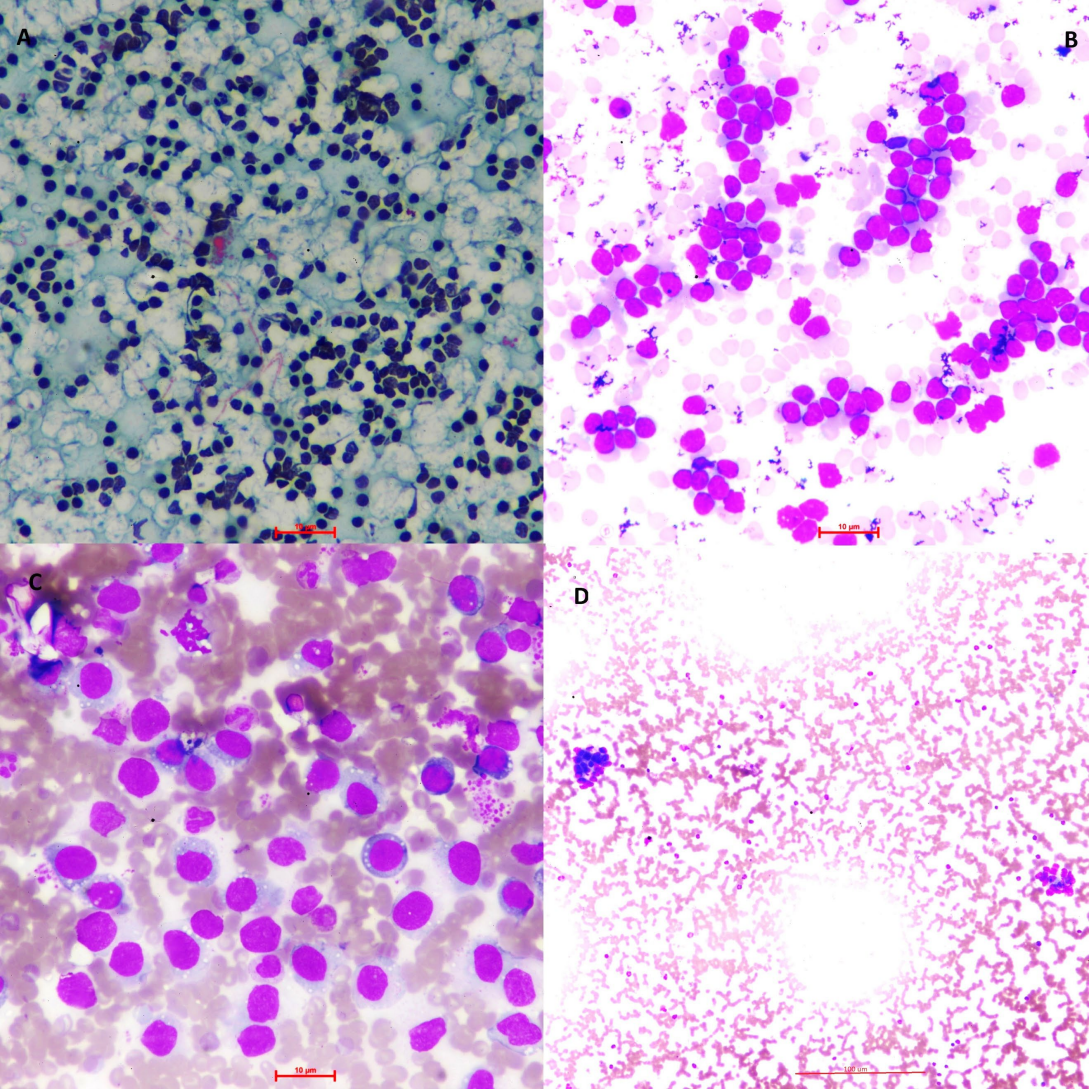
A: Microphotograph shows monomorphic population of atypical lymphoid cells. AUS category.(Pap, 10x) B: Microphotograph shows monomorphic population of atypical lymphoid cells. Suspicious category.(Giemsa, 40x) C: Microphotograph shows monomorphic population of medium sized lymphoma cells. Malignant category. (Giemsa, 40x) D: Microphotograph shows tumor cells arranged in vague acini. Suggestive of Metastatic adenocarcinoma. Malignant category. (Giemsa, 10x)

**Table 4.** Sensitivity, specificity, positive predictive value, negative predictive value, and accuracy of lymph node FNAC.

Statistics	Value	95% CI
Sensitivity	66.67%	40.99% to 86.66%
Specificity	85%	62.11% to 96.79%
Positive predictive value	80%	57.27% to 92.27%
Negative predictive value	73.91%	58.97% to 84.82%
Diagnostic Accuracy	76.32%	59.76% to 88.56%

**Figure:**
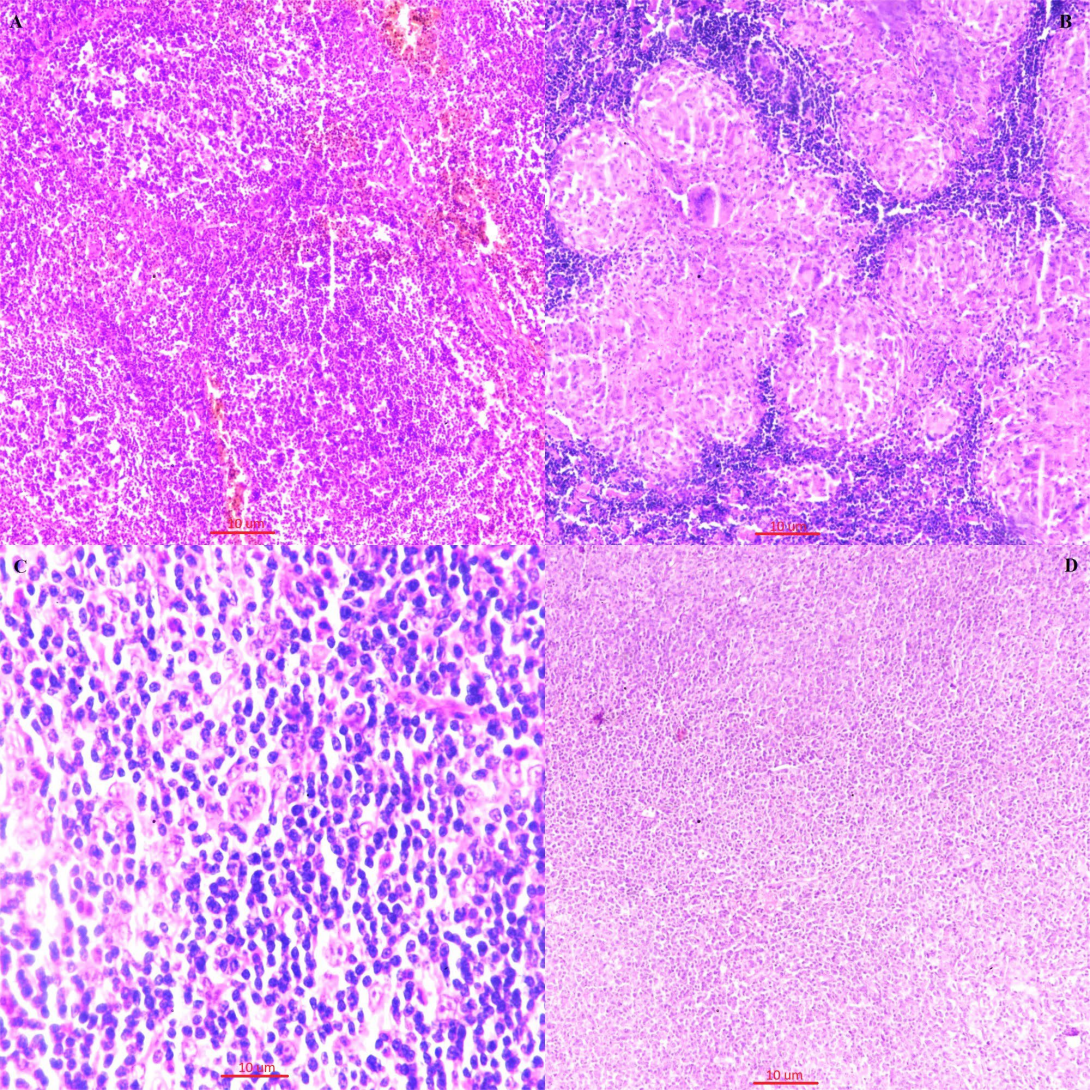
A: Microphotograph shows reactive lymphoid follicles of varying sizes. Features are of reactive lymphoid hyperplasia. (H&E, 4x) B: Microphotograph shows well-formed granuloma. Features are of granulomatous lymphadenitis. (H&E, 10x) C: Microphotograph shows mononucleated and binucleated RS cells. Features are of Hodgkin’s lymphoma. (H&E, 40x) D: Microphotograph shows monomorphic population of small sized lymphoma cells. Features are of Non-Hodgkin’s lymphoma. (H&E, 10x)

**Table 5:** The risk of malignancy for individual Sydney category in various studies

Study	L1 (inadequate)	L2 (benign)	L3 (AUS)	L4 (Suspicious)	L5 (Malignant)
Gupta et al (2021) [9]	27.5%	11.5%	66.7%	88%	99.6%
Vigliar et al (2021) [12]	50%	1.92%	58.3%	100%	100%
Caputo et al (2021) [13]	66.7%	9.38%	28.6%	100%	99.8%
Makarenko et al (2021) [14]	58.3%	6.4%	69.2%	96.7%	99.3%
Present study	25%	5.2%	50%	80%	88.8%

## Discussion

Lymph node cytology can be challenging. However, with proper handling of diagnostic material, the use of special stains and ancillary techniques, and integration of clinical data, satisfactory diagnostic accuracy can be achieved. In our study, we demonstrated a high level of diagnostic accuracy. Although a standardized reporting system has been lacking, FNAC of lymph nodes continues to be widely used and trusted by clinicians.

In other areas of cytopathology, standardized reporting systems such as Bethesda, Milan, and Yokohama have been adopted successfully, facilitating clear communication of clinically relevant information with minimal interobserver variability (10). These systems also provide management recommendations for each diagnostic category, reducing the likelihood of misinterpretation (11). For lymph node cytology, risk stratification with corresponding ROM values is essential for establishing reliable and reproducible diagnostic categories.

In a study by Vigliar et al (2021), 300 cases of lymph node FNAC were analyzed, with patient ages ranging from 13 to 85 years. Cervical lymph nodes were the most commonly affected, consistent with our findings (12). Makarenko et al reported 41% female patients in their series, which was also concordant with the present study (13). Caputo et al documented a sensitivity of 97.9%, specificity of 96.9%, positive predictive value of 99.6%, and negative predictive value of 86.3%, results broadly comparable to our observations (14). Ahuja et al conducted the first meta-analysis to evaluate pooled ROM values for the Sydney System. This analysis included nine retrospective cross-sectional studies with a total of 13,205 cases, demonstrating the accuracy and clinical relevance of the Sydney System in reporting lymph node aspirates (15). Very few studies have calculated ROM across categories; our results are comparable with those of Gupta et al (9).

In the present study, ROM for the L1 (nondiagnostic) category was 25%. Most cases in this group involved subcentimetric or deep-seated lymph nodes, such as mesenteric or periportal nodes, and yielded only blood or insufficient material. Repeat FNAC, preferably ultrasound-guided or with ROSE, is advisable in such cases. Our findings support the Sydney System recommendations, which include repeat FNAC, core-needle biopsy, or excisional biopsy depending on the clinical context (16). One discordant case in this group involved metastatic malignant melanoma missed on FNAC, likely due to technical error. Repeat aspiration with ROSE by an experienced cytopathologist could have prevented this misdiagnosis.

The L2 (benign) category comprised the largest proportion of cases, as reported in all studies. Reactive lymphadenitis was the most common diagnosis, followed by granulomatous lymphadenitis. One discordant case involved peripheral T-cell lymphoma misdiagnosed as Kimura disease. Retrospective review showed polymorphous background and scattered atypical lymphoid cells, indicating an interpretative error. Such false negatives may be avoided by repeat FNAC in clinically suspicious cases. ROM values in this group were consistent with other published series. Our findings also aligned with Sydney System management guidelines, which recommend clinical follow-up when cytological, radiologic, and clinical features are concordant, and repeat FNAC with immunophenotyping when discordance exists (17).

The L3 (AUS) category had a ROM of 50%. Seven discordant cases were noted, three of which were misreported as reactive lymphoid hyperplasia. Retrospective review revealed polymorphous lymphoid populations with immunoblasts misinterpreted as Reed-Sternberg–like cells. Other cases suffered from poor smear quality and hemorrhagic background, obscuring atypical lymphoid cells. Many of these cases should have been categorized as “suspicious for malignancy,” which would have improved concordance. Repeat FNAC, core-needle biopsy, or excisional biopsy, ideally with flow cytometry or cytogenetics, is recommended for this category, as emphasized by the Sydney System (17).

In the L4 (suspicious) category, five cases had histopathological follow-up, with an ROM of 80%. One discordant case was initially interpreted as suspicious for metastatic papillary thyroid carcinoma but was later confirmed as reactive lymphoid hyperplasia on biopsy. Sampling error during ultrasound-guided core biopsy likely contributed to this misclassification. Complete excisional biopsy would have been preferable in this setting. Our findings support the Sydney System recommendation of repeat FNAC, CNB, or excision biopsy for this category (18).

The L5 (malignant) category had the highest ROM at 88.8%. One discordant case involved a five-year-old boy in whom non-Hodgkin lymphoma was misdiagnosed on FNAC, but histopathology revealed reactive lymphoid hyperplasia. The smear was paucicellular with a predominance of small lymphocytes, leading to overinterpretation. This underscores the diagnostic pitfalls of lymphoid cytology in pediatric patients.

The main limitations of our study were the small number of histopathological follow-up cases and the relatively short study period. Most FNAC cases were benign and did not undergo biopsy, which may have impacted ROM calculations. Another limitation was the lack of immunocytochemistry, as cell blocks were not prepared. Larger multicenter studies with longer follow-up and use of ancillary techniques such as immunohistochemistry are needed to validate the reliability and reproducibility of the Sydney System.

## Conclusion

The present study demonstrated an overall diagnostic accuracy of 76.3%, highlighting the effectiveness of the Sydney System in lymph node FNAC. Structured reporting using this system improved uniformity, accuracy, and reproducibility of diagnoses. Despite limitations related to histopathological follow-up and ancillary testing, risk-based stratification with the Sydney System facilitated better communication of ROM for each diagnostic category, thereby supporting informed clinical decision-making and guiding appropriate patient management.

## Data Availability

All data generated during the study are included in this article. Further enquiries can be directed to the corresponding author.
